# Varicose veins and occupational health: symptoms, treatment and prevention

**DOI:** 10.5327/Z1679443520190460

**Published:** 2019-12-01

**Authors:** Denis Camargo de Lima

**Affiliations:** 1 Ribeirão Preto School of Medicine, Master’s in Health Organization Management - Ribeirão Preto (SP), Brazil. Ribeirão Preto School of Medicine Master’s in Health Organization Management Brazil

**Keywords:** venous insufficiency, occupational medicine, health, varicose veins

## Abstract

The aim of the present study was to perform a literature review about the symptoms, treatment and prevention of varicose veins (VV) within the occupational medicine setting. I reviewed scientific articles, books, master’s and doctoral dissertations and synthesized the results of quantitative and qualitative studies. I further retrieved information from Brazilian federal government occupational health websites. The time frame considered was the period from 2004 through 2018. VV are abnormally dilated, twisted and congested veins caused by prolonged peripheral venous hypertension and chronic venous insufficiency. VV most commonly involve the lower limbs in association with static posture and continuous contraction which exhaust the muscles, especially among individuals who remain standing over long periods of time. VV are associated with risk factors such as obesity, sedentary lifestyle and hormones. Symptoms include feelings of tiredness, pain and swelling. When untreated VV might result in venous ulcers. Occupational physicians should promote changes in the workers’ lifestyle, particularly as concerns physical activity (stretching and walking), local massage and elevating the lower limbs - feet about 15 cm above the heart level, and prescribe compression stockings or bandages, and medications such as diosmin, calcium dobesilate, rutosides and horse chestnut extract.

## RESUMO |

Este estudo teve o objetivo de apresentar uma revisão de literatura sobre os sintomas, o tratamento e a prevenção das varizes na saúde do trabalhador. Para tanto, realizou-se uma revisão de artigos científicos, livros, dissertações e teses, por meio da síntese de resultados de estudos quantitativos e qualitativos, e de *sites* do governo federal relacionados à saúde do trabalhador e à medicina do trabalho, compreendendo o período de 2004 a 2018. Entendeu-se que as varizes são sinais caracterizados por veias com dilatação anormal, tortuosa e congestionada, ocasionadas pela hipertensão venosa prolongada e periférica e insuficiência venosa crônica (IVC). Comumente evidenciada nos membros inferiores, por causa da postura estática e da contração contínua que fadigam os músculos, principalmente em indivíduos que permanecem em pé por muito tempo, a ocorrência de varizes associa-se a fatores de risco como obesidade, sedentarismo, estilo de vida e de trabalho e hormônios. Como sintomas, apresentam-se cansaço, dor e inchaço, podendo, se não tratada a varize, provocar úlceras varicosas. Verificou-se que a medicina do trabalho deve promover mudança de hábito de vida dos trabalhadores, sobretudo atividades físicas (alongamento e caminhada), massagem local, descanso com os pés aproximadamente 15 cm acima do nível do coração, indicação de utilização de meias compressivas ou de bandagens funcionais e de medicamentos como a diosmina, o dobesilato de cálcio, os rutosídeos e o extrato de castanha-da-índia.

## INTRODUCTION

As is known, work has a double paradoxical nature: it is a source of accomplishment, pleasure and satisfaction, and thus is one of the grounds of the process of development of personal identity. However, it might also become pathogenic, i.e. harmful to health. Indeed, routine occupational activities might include physically exhausting components which largely determine the type and organization of work^1^.

Among work-related diseases, chronic venous insufficiency (CVI) in the lower limbs - the main cause of varicose veins (VV) - in mild degree affects up to 80% of the global population, while intermediate and severe cases represent 20% to 64% and 9%, respectively^2^. The etiology of CVI is associated with some lifestyle aspects, such as body posture and standing over long periods of time at work^3^.

Sustaining static standing requires continuous, low-intensity muscle tension. Prolonged muscle contraction compresses the blood vessels, with consequent impairment of the blood and lymphatic circulation. This condition causes several disorders in the lower limbs, among which VV stand out^4^.

A result of abnormal vein function due to valve insufficiency associated or not with flow obstruction, VV have considerable socioeconomic impacts derived from medical, hospital and social security costs^5^^,^^6^. According to the Brazilian National Social Security Institute (INSS)^7^ lower extremity VV accounted for 42,899 sick-pay benefits from January through December 2016 in the country.

As a function of the considerable morbidity associated with VV leading to impaired productivity, retirement and restrictions in activities of daily living and leisure, multidisciplinary and occupational medicine scientific studies on how the symptoms, treatment and prevention of VV are characterized within the context of occupational health are relevant. Therefore, my aim in the present study was to perform a literature review to contribute to the understanding of the pathophysiology of VV and characterize treatment and prevention within the occupational medicine setting.

## METHODS

I had resource to a scientific deductive (from the general to the particular) approach to contribute to the understanding of the symptoms, treatment and prevention of VV among workers, since CVI causes several problems, including swelling and ulcers. My focus was on the prevalence of VV, as is known, a frequent occurrence among workers, especially those who must spend long periods of time standing.

The time frame for the search was set from 2004 to 2018. I considered books, scientific articles and dissertations cited in electronic databases PubMed/MEDLINE, Scientific Electronic Library Online (SciELO), Latin American and Caribbean Health Sciences Literature (LILACS) and Regional Library of Medicine (BIREME). The search terms used were *venous insufficiency, occupational medicine, health* and *varicose veins*.

## PATHOPHYSIOLOGY OF VARICOSE VEINS

VV are abnormally dilated, twisted and congested veins which appear as a result of prolonged venous hypertension. While any vein in the body might be involved, the superficial veins of the lower limbs are the most commonly affected in association with standing over long periods of time at work^8^^,^^9^.

A static posture significantly interferes with the peripheral circulation, since continuous contraction of some muscles causes fatigue and impairs the venous return from the lower limbs^4^^,^^10^. Venous reflux has a relevant role in the origin of the signs and symptoms of lower extremity CVI, which range from feelings of tiredness to infection of extensive chronic ulcers^8^^,^^11^.

VV might affect the superficial, deep or both types of veins. Disorders might be congenital or acquired, and several risk factors are associated with occurrence of venous insufficiency, particularly obesity, age, sex, sedentary lifestyle, work, diet, use of hormones and pregnancy^5^^,^^11^.

In the case of primary VV associated with focal or generalized vein wall dysfunction, dilation causes valve insufficiency whereby the cusps are pushed apart^2^. Primary VV occur in superficial veins, while secondary VV involve the deep ones leading to vein malformation, arteriovenous fistula, deep vein thrombosis, venous trauma and occlusion^8^. According to another commonly used classification system, affected veins are categorized as reticular or varicose^6^^,^^12^. The reticular are bluish, subdermal twisted veins with 1 mm to 3 mm of diameter, to the exclusion of visible veins among people with thin and translucent skin. In turn, varicose are subcutaneous, dilated and also twisted veins with over 3 mm of diameter while standing and correspond to the great saphenous vein, its tributaries or unrelated lower extremity superficial veins^12^.

When veins dilate, the blood exerts increasingly higher hydrostatic pressure, which is transmitted to the capillaries, with consequent leak of fluid and small proteins to the extravascular space. Initially, this effect is compensated throughprotein and lymph reabsorption^10^. However, as venous hypertension becomes chronic, compensation becomes insufficient and swelling appears. Edema, which develops as an inflammatory response involving neutrophils, phagocytes and macrophages, increases the capillary permeability whereby extravasation ensues including larger blood components, such as red cells. From the immune perspective, as a result of the attempts to reabsorb macromolecules, macrophages develop cytoplasmic granules containing free radicals, which potentiate the inflammatory response^2^. Progressive increase of the interstitial pressure impairs the oxygen transfer and metabolic exchanges, which result in red blood cell lysis. Free hemoglobin is associated with production of hemosiderin, which is irritant to tissues. The skin dries up, causing thinning and desquamation, increased oncotic activity and fibrosis, which phenomena define the stage of venous ulcer^2^^,^^11^. The most severe form of CVI is characterized by ulcers and inflammatory lesions which usually demand conventional surgical treatment^13^.

## VARICOSE VEINS AND OCCUPATIONAL MEDICINE

As a function of the stage in the progression of CVI and the severity of symptoms, occupational physicians may need to refer patients to an angiology specialist. Appropriate clinical assessment of lower limb venous insufficiency allows detecting the involved vein systems or anatomical levels and to determine the need for invasive or noninvasive diagnostic tests^14^.

Invasive tests, such as phlebography and ambulatory vein pressure monitoring, afford an accurate diagnosis, but are uncomfortable and are not free from complications. Noninvasive tests, including Doppler ultrasound, photoplethysmography, air plethysmography and duplex venous mapping, provide anatomical and functional information and enable the assessment of the deep and superficial venous systems^14^.

A study of the prevalence of risk factors for CVI among humanities professors in a private university pointed to the need to assess these factors separately according to sex, time spent in static posture, excess weight and use of hormones^5^.

The authors of a study conducted to analyze the lower limb volume among 20 health care providers at a hospital in Maringá, Paraná, Brazil, found that swelling occurred continuously along the working hours^15^. Within the occupational medicine setting, swelling and VV may be prevented or controlled through adequate mechanical or pharmacological prophylaxis^16^.

Lifestyle changes should be promoted, especially through the inclusion of exercise to strengthen the calf muscles, such as walking, and thus normalize the venous return. High heels should be avoided, since use interferes with the hemodynamics of the sole and calf muscle pump. As indicated within preventive medicine, combating obesity, physical activity and elevating the legs contribute to normalize the venous return. Elevating the feet to about 15 cm above the heart level in alternation with walking was found to reestablish the normal lower limb hemodynamics^16^.

The relevance of physical activity in CVI was demonstrated in a study performed at School of Surgery, Federal University of Minas Gerais, and University of São Paulo, Brazil, with 100 individuals above age 50 from both sexes. Exercise - walking, running, cycling, swimming - was associated with lower rates of CVI compared to sedentary individuals and less progression of disease into ulcers^17^.

In a study with salespeople, VV were detected in half of those who worked standing over a long period of time (12 hours). As also found in other studies, occupational medicine interventions should promote continuous movement to improve the venous circulation and reduce the time of standing work. Intervals of at least 15 minutes sitting on armchairs and elevating the feet might contribute to normalize the lower limb venous flow^8^.

A study performed at an angiology clinic in Vitória da Conquista, Bahia, Brazil, confirmed the occurrence of definite risk factors in the workplace, among which, working in the same position over long periods of time stood out, involving 77% of the workers^18^. Another study conducted in this city detected lower limb VV in 36.1% of 250 teachers from 10 different schools^1^.

Occupational physicians should also encourage stretching, since it serves for warming up, as is shown in the literature^19^. Equally relevant are massage and passive movements performed by physical therapists to improve the lower limb venous homeostasis. Soft and firm centripetal massage of the skin and subcutaneous tissue using hydrating oils and ankle movements were proven to be beneficial^16^.

One further measure widely recommended by specialists is wearing compression stockings, which by helping control venous reflux is efficacious for individuals with CVI. In addition, by improving the venous and lymphatic drainage, this method improves the microcirculation and increases the venous return velocity, with consequent reduction of venous and capillary stasis, which represents the main therapeutic target in CVI^16^^,^^20^.

In a study with seven nursing technicians aged 21 to 41 years old working 6 hours/day in the afternoon at a quaternary hospital surgery department, wearing compression stockings helped reduce lower limb swelling and symptoms including pain, heaviness and feelings of tiredness^21^. However, indication should be made cautiously for patients with chronic occlusive peripheral artery disease, infection, congestive heart failure and advanced peripheral neuropathy^22^.

One further issue regarding compression stockings is the choice of elastic or inelastic forms. Inelastic compression is effective to reduce swelling only during muscle contraction and relaxation movements. In turn, elastic compression might be adjusted as a function of changes in the lower limb volume and affords continuous pressure to the skin^16^. Graduated compression stockings should be indicated according to the aim pursued, i.e. prevention or therapeutic treatment, respectively under or above 15 mmHg, as shown in a study performed at Mackenzie Presbyterian University with 40 traffic enforcement agents in Barueri, São Paulo, Brazil. The authors further found that also functional bandages may be used for this purpose, which are applied as stockings, but afford lower pressure levels and thus enable a wider range of pressure variation to the tissues and freedom of movement^23^.

Occupational physicians may also consider prescribing complementary medications, such as diosmin, calcium dobesilate, rutosides and horse chestnut extract, which induce considerable swelling reduction^23^. However, these drugs should not be seen as a replacement for compression therapy and the lifestyle changes necessary to improve venous stasis, or surgery when properly indicated by vascular surgeons^24^^,^^25^.

## CONCLUSION

Venous reflux caused by chronic, prolonged and peripheral lower limb venous hypertension accounts for CVI symptoms, such as swelling, pain and feelings of tiredness, and patent signs, including VV and ulcers in the most severe cases. Impacts on the quality of life of workers and socioeconomic effects, medical, hospital and social security costs strongly point to the need for occupational physicians to promote changes in the workers’ lifestyle, mainly in regard to physical activity (stretching and walking), local massage, elevating the lower limbs while at rest - feet about 15 cm above the heart level, compression stockings and bandages, and medications such as diosmin, calcium dobesilate, rutosides and horse chestnut extract. Preventive and therapeutic strategies should be devised targeting problems in the workplace related to CVI based on a multidisciplinary approach that considers clinical aspects and personal and environmental risk factors.
